# Supplementary material for: Association of CARD14 Single-Nucleotide Polymorphisms with Psoriasis
Source: Int J Mol Sci. 2022 Aug 19;23(16):9336. doi: 10.3390/ijms23169336 (PMC9409305; doi:10.3390/ijms23169336)
Supplement: Supplementary file 1 [file ijms-23-09336-s001.zip › ijms-1864134-supplementary.pdf]

**Supplementary Table S1.** Primers used to amplify the CARD14 regions to genotype the selected SNPs.

| No. | Primer | Sequence | Tm | Length | Hits | Product |
|---|---|---|---|---|---|---|
| **1** | Forward | CTGGATTTGCTGAAGACTCG | 56.5 | 20 | 1 | |
| | Reverse | ATCCAACCCCAATACAGTGA | 56.1 | 20 | 1 | 310 |
| **2–10** | Forward | CCTGCCCACCTATTACCTC | 57.1 | 19 | 1 | |
| | Reverse | GGCAGGGAAGTGTTAGACAA | 57.8 | 20 | 1 | 504 |
| | Reverse | ATTGCTATAGTGCAGCGAGA | 56.4 | 20 | 1 | 330 |
| **11** | Forward | ATCAAGGTCCCAAGCATTG | 58.5 | 19 | 1 | |
| | Reverse | ATAGGACACTCACCCTCTGC | 56.7 | 20 | 1 | 392 |
| | Reverse | GATGAAGATGCCCGTGAG | 57.6 | 18 | 1 | 674 |
| | Reverse | GACCCGGTGGATGAAGAT | 58.2 | 18 | 1 | 683 |
| **12–15** | Forward | GCAGAGGGTGAGTGTCCTAT | 56.7 | 20 | 1 | |
| | Reverse | ATGAGCAGAACGCTTTACG | 56.7 | 19 | 1 | 549 |
| | Reverse | ACGGCACGACTCTCATCTAT | 57.3 | 20 | 1 | 527 |
| **16** | Forward | GCAGAGGCTCGTATCTGTG | 57.0 | 19 | 1 | 263 |
| | Forward | GACGGTACACATACCACTCC | 55.3 | 20 | | 525 |
| | Reverse | GGCAGTATCCTTCATGGTG | 56.4 | 20 | 2 | |
| **17** | Forward | GCCAATCATCTCCCCTGA | 59.5 | 18 | 1 | |
| | Reverse | GCCTAAATGAGTGGCATCC | 58.1 | 19 | 1 | 304 |
| **18** | Forward | CTATGGAACTGGGAGTGAGTC | 56.3 | 21 | 1 | |
| | Reverse | CACAGTTTCTCGCTCAGGAT | 58.0 | 20 | 1 | 305 |
